# Factors Affecting Synthetic Dye Adsorption; Desorption Studies: A Review of Results from the Last Five Years (2017–2021)

**DOI:** 10.3390/molecules26175419

**Published:** 2021-09-06

**Authors:** Eszter Rápó, Szende Tonk

**Affiliations:** 1 Environmental Science Department, Sapientia Hungarian University of Transylvania, Calea Turzii No. 4, 400193 Cluj-Napoca, Romania; 2 Department of Genetics, Microbiology and Biotechnology, Hungarian University of Agriculture and Life Sciences, Páter Károly No. 1, H-2100 Gödöllő, Hungary

**Keywords:** synthetic dyes, historical briefing of dye usage, adsorption influencing parameters, desorption eluents

## Abstract

The primary, most obvious parameter indicating water quality is the color of the water. Not only can it be aesthetically disturbing, but it can also be an indicator of contamination. Clean, high-quality water is a valuable, essential asset. Of the available technologies for removing dyes, adsorption is the most used method due to its ease of use, cost-effectiveness, and high efficiency. The adsorption process is influenced by several parameters, which are the basis of all laboratories researching the optimum conditions. The main objective of this review is to provide up-to-date information on the most studied influencing factors. The effects of initial dye concentration, pH, adsorbent dosage, particle size and temperature are illustrated through examples from the last five years (2017–2021) of research. Moreover, general trends are drawn based on these findings. The removal time ranged from 5 min to 36 h (E = 100% was achieved within 5–60 min). In addition, nearly 80% efficiency can be achieved with just 0.05 g of adsorbent. It is important to reduce adsorbent particle size (with Φ decrease E = 8–99%). Among the dyes analyzed in this paper, Methylene Blue, Congo Red, Malachite Green, Crystal Violet were the most frequently studied. Our conclusions are based on previously published literature.

## 1. Introduction

Over the centuries, human ambition and the desire for comfort have brought with them the degradation of the natural environment. This has led to a deterioration in air quality, over-exploitation of soils and their barrenness through inappropriate management, and left our natural waters heavily polluted—a problem that needs to be solved [1].

Between 2000 and 2020, the global population increased from 6.1 billion to 7.8 billion people. During this period, 2 billion people gained access to safely managed drinking water services, and the number of people lacking safely managed services decreased by 342 million [2]. The rapid population growth is leading to agricultural and industrial overproduction, with a concomitant decline in water quality and a reduction in quantity as well. One of the causes of the freshwater crisis, which is slowly unfolding worldwide, is the presence of various natural or man-made contaminants [3]. As a result of the development of human civilization, the pollution caused by the release and/or use of a wide range of chemicals has reached serious proportions. Global anthropogenic pollution has led to the accumulation of a wide range of organic xenobiotic compounds that have adverse effects on human health and intact ecosystems. Xenobiotics are compounds that do not exist as natural products or may contain structural elements that cannot be synthesized biochemically [4].

Pesticides, pharmaceuticals, heavy metals, oils, detergents, industrial chemicals and dyes can reduce taste quality. The sources of dye contaminants in freshwater can be the textile, pharmaceutical, food, leather, paint and varnishing industry effluents. Other sources are households, and moreover the untreated or partially treated effluents from wastewater treatment plants [3]. According to the literature, five major industries are known to be responsible for the presence of dye effluents in the environment: the textile industry (54%), the dyeing industry (21%), paper and pulp industry (10%), tannery and paint industry (8%), and the dye manufacturing industry (7%) [5,6].

After the dyeing process of textiles, the resulting dye-concentrated wastewater is often discharged into nature at high pH and temperatures without any treatment. The oxygen transfer mechanism and the self-purification process of environmental water bodies will get disturbed by this phenomenon [5,7,8]. Wastewater from the paint industry is a difficult effluent to treat, not only because of its high biological and chemical oxygen demand, high suspended solids content and other hazardous substances, but also because of the aesthetic harm it causes to the visual appearance [9,10]. These substances are often of synthetic origin and have a complex aromatic molecular structure, which increases their chemical and microbiological stability, hence their difficult removal from water. The introduction of dyes into the water system causes a number of health and environmental problems:

- Dyes increases the water turbidity;
- Dyes have a major impact on the photosynthetic activity of the aquatic environment because they block the penetration of light into the water, thus inhibiting the growth of algae, which are not only important for oxygen production but are a pillar of the food chain;
- Most of the dyes are carcinogenic (bladder, kidney, liver), mutagenic and toxic to living organisms;
- They can cause allergic reactions: skin, eye, mucous membrane irritation, dermatitis, respiratory problems; and
- They cause harm to aquatic environment, and may be toxic to aquatic organisms due to their aromatic, heavy metal and chlorine content [3,11,12].

The presence of dyes in natural waters has not received attention in the last 30 years, and has only recently become part of environmental legislation. As per this law, dye utilizing industries have to ensure wastewater released from their factories abide by the International Dye Industry Wastewater Discharge Quality Standards that were adopted from the Zero Discharge of Hazardous Chemicals Programme (ZDHC) [5,13].

The aim of this review article is to provide up-to-date information on the adsorption of dyes from aqueous solutions, highlighting the parameter influencing processes. As to the best of our knowledge, there is a niche in articles that summarize this aspect from the last five years (2017–2021). The focal aim of this paper is to review the effects of initial dye concentration, pH, adsorbent dosage, particle size and temperature through examples from the last five years of research. Moreover, general trends are drawn based on these findings. In addition, different definitions of dyes are presented at the beginning of the article, with a brief overview of the historical background and the numerical, statistical data of their usage and application. The general structure and classification methods are also described. Finally, the eluents used for adsorbent regeneration and desorption are listed, and desorption examples are presented.

### 1.1. Definition of Dyestuff

Dyestuffs are hydro or oil-soluble, colored organic chemical compounds that are usually dissolved in water and bound to surfaces or fabrics to impart color to textiles. The majority of dyes are complex organic molecules that are designed to bind strongly to the polymer molecules that make up the textile fiber, and must be able to withstand a wide range of external effects [14,15,16].

In his book “Synthetic dyes”, Gurdeep R. Chatwal [17] defines dyes as colored organic compounds or mixtures used to color paper, cloth, plastics and leather. The dye substrate must be resistant to washing and stable to light. It is important to note that not all colored materials are dyes, as a dye must be fixed to the material to give it a permanent color [17].

According to the internationally accepted convention of Colour Index International, dyes are defined as intensely colored or fluorescent organic substances that impart color to a substrate by selective light absorption. These substances dissolve and/or undergo a process that destroys, if not permanently, the crystal structure by adsorption, mechanical action, ionic or chemical bonding [18].

Dyes are usually large aromatic molecules, often with many rings linked together. An aromatic ring structure linked to a side chain in the dye molecule structure is necessary for resonance and hence for the transfer of color [19]. The resonance structures responsible for color are those that cause the shifting or appearance of absorption bands in the visible spectrum of light. In the synthesis of a dye, the correlation of chemical structure and color is achieved by a chromogen-chromophore-auxochrome combination. Three essential groups can be found in a dye molecule: the chromophore, auxochrome and matrix [16]. Thus, dyes are organic colorants that contain at least one unsaturated compound (chromophores) and one functional group (auxochromes). The chromophore present in the structure may be an aromatic structure containing benzene, naphthalene, or anthracene rings. The chromophore group responsible for the color formation is represented by the following radicals: azo (-N=N-); carbonyl (=C=O); carbon (=C=C=C=); carbon-nitrogen (>C=NH or -CH=N-); nitroso (-NO or N-OH); nitro (-NO or =NO-OH); and sulfur (>C=S, and other carbon-sulfur groups). These, in combination with a chromogen, form the basis for the chemical classification of dyes. Since the chromogen-chromophore structure is often insufficient to provide adequate solubility and thus the dye cannot adhere to the fiber of the material, auxochromes are required. Auxochromes enhance the color of the dye. Auxochromes, also known as binding affinity groups, can be amine (-NHX_2_), hydroxyl (-OH), carboxyl groups (-COOH), aldehydes (-CHO), sulfonic acid (-SO_3_H) or their derivatives [20,21,22,23].

### 1.2. Brief History of Dye Usage

The word dye is from Middle English “deie” and from Old English “dag” and “dah”. The first known use of the word dye was before the 12th century [24].

**Figure 1:**
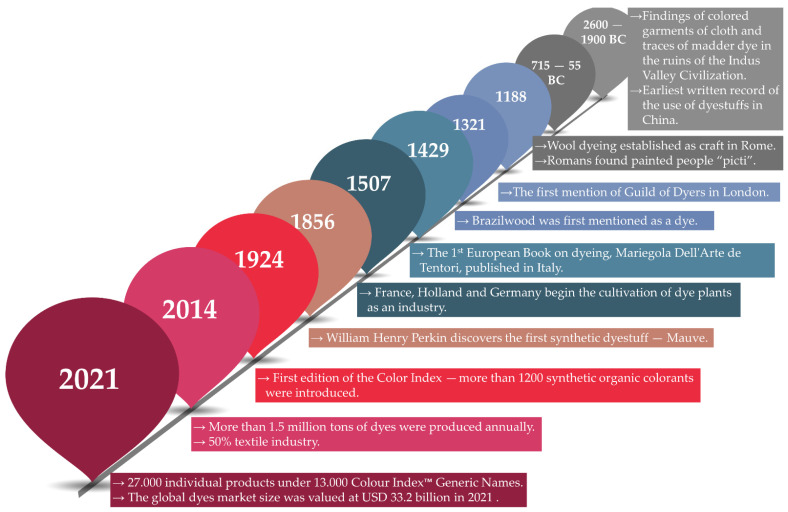
Historical timeline of dye usage, invention and interesting facts.

Human eyes can see more than one million colors, all of which can be found in our natural habitats. These wonderful and unique colors attract humans’ attention from the surroundings, and everyday tools were made to mimic these colors. Archeological excavations prove that the art of dying can be dated back to the appearance of human civilization. Figure 1 contains a timeline, based on the detailed historical overview of Susan C. Druding (unfortunately, the literature data has been lost, so its references are missing), where some important historical milestones regarding dyestuffs are represented [25,26].

Without wishing to be exhaustive, we would like to mention a few interesting facts, as a detailed list can be found in the literature. According to these data, colored garments of cloth and traces of madder dye were found in the ruins of the Indus Valley Civilization dated between 2600 and 1900 BC. Moreover, the first written record about dyestuff usage was found in China during this period [27]. Another interesting investigation showed that the cave paintings of “El Castillo” in Spain were painted about 40,000 years ago. Probably the oldest colored flax fiber dated around 34,000 BC was found in the Republic of Georgia (in a prehistoric cave) [28]. Several mentions are made between 715 and 55 BC, from the Roman Empire, where wool dyeing appeared as a craft, and purple has been used for dyeing their clothing, like robes. After the conquest of Susa in 333 BC (the capital of Persia), Alexander the Great mentions that he found purple cloths in the royal treasury (dating from 541 BC) [24,25,26]. The 5000 talents of purple cloth colored with mucus (yellowish material from sea snail’s tiny gland near its neck) today is worth about $68 million [29].

Jumping ahead in time, the 12th century saw the establishment of several painters’ guilds in Europe’s major cities (e.g., London in 1188). In Florence, in the middle of the century, there were more than 200 registered painters, clothiers and tailors. Several rulers took measures to protect merchants and quality [25].

At the beginning of the 15th century, Cennino Cennini (Padua, Italy) published his treatise, the Method of Painting Cloths by Means of Moulds, in which he described the method of printing cloth. The first European book on painting, Mariegola Dell’Arte de Tentori, was published in Italy in 1429. From 1507 onwards, several European countries (France, the Netherlands and Germany) began to grow dye plants on an industrial scale [25].

Prior to the industrial revolution, to the middle of the 19th century, all dyestuff was made from natural sources: plants, animals, and minerals. The small quantities of the main components of dyes, the long distances involved, and the weather conditions were the economic disadvantages of using natural dyes. For this reason, there was a need to be able to produce commonly used dyes quickly and easily by synthetic means in any region, thus making the product cheaper, and transport and trade more reliable. Literature records suggest that the substitution and thus production of naturally occurring indigo and madder dyes posed difficulties for chemists of the time [29].

The root of the *Rubia tinctorum* plant, most commonly cultivated in Turkey, was used to extract cadherin, whose coloring principle is alizarin. In a complicated process, it was mixed with aluminum to form an insoluble red metal complex, bright red in color, with cellulose fibers.

Indigo, also a plant dye (*Indigofera tinctoria*), was the most important natural blue dye. In ancient times, the flowering indigo plant was cut and fermented in wooden vats underwater for 10–15 h. A yellow solution was obtained, from which the raw indigo was released as blue flakes in the air. The leaves of the plant are rich in indoxyl, and after fermentation, free indoxyl is released, which is rapidly oxidized in air to the desired color, and is insoluble in water [29].

Therefore, the discovery and development of synthetic dyes are closely intertwined with the development of organic chemistry and the industrial, economic, and social demands of the 19th century. There were a lot of attempts to produce synthetic dyes; however, these were not successful due to their poor lightfastness. The discoverer and pioneer of synthetic dyes is said to be William Henry Perkin. On Easter 1856, while studying the production of artificial quinine for the treatment of malaria (oxidized dichromate), he isolated a small amount of purple dye. He named the dye ‘mauve’, which soon became a favorite of the royal family, and a new industry was launched [30]. Until the beginning of the twentieth century, the dye industry continued to flourish, with many different types of dyes being produced, making it essential to classify, record and catalogue them. In 1924, the first edition of the Color Index was published, listing over 1200 organic and synthetic dyes.

It was reported that in 2014, more than 1.5 million tons of dyes were produced worldwide, out of which 50% were used by the textile industry [31,32]. According to an article published in 2016, over 50,000 tons of different synthetic dyes were annually produced and approximately up to 10% were mixed with water bodies [33].

Up to date statistics show that the global dyes market size was valued at USD 33.2 billion in 2021. The Colour Index™ contains 27,000 individual products under 13,000 generic names and properties [34,35]. It is projected that the revenue generated by the manufacture of dyes and pigments in Romania will amount to approximately $65.1 million by 2023 [36].

### 1.3. Classification of Dyes

**Figure 2:**
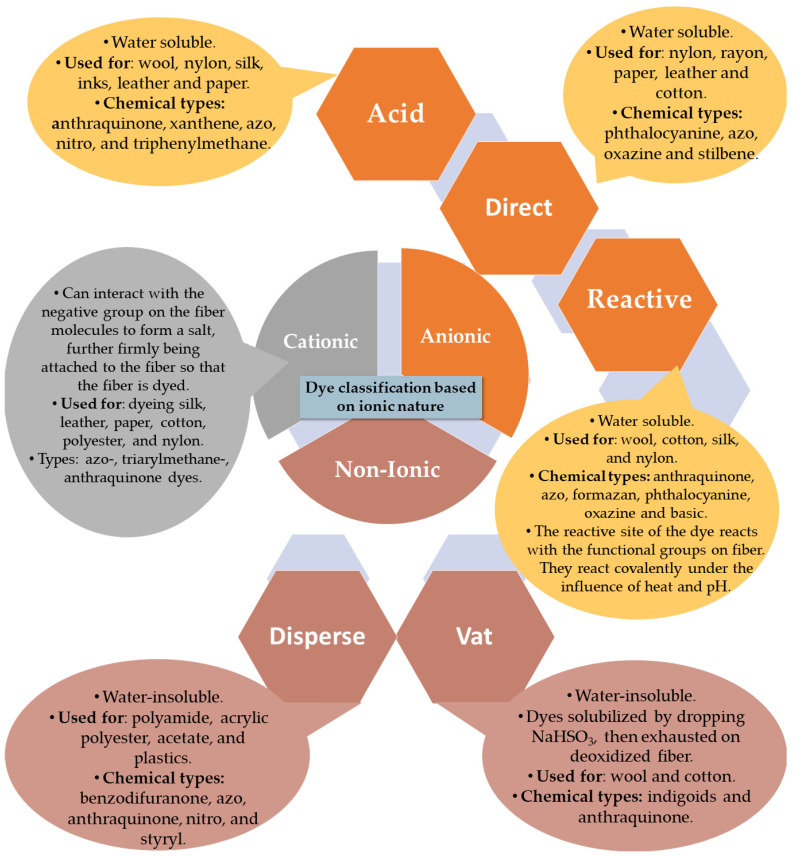
Dye classification based on ionic nature.

As the quantity and variety of dyes has increased throughout history, it has become essential to classify them. There are several different classifications, based on their structure, source, color, solubility and application methods. Basically, the most common classification is based on their chemical structure and application [37]. Figure 2 combines the grouping by ionic nature (particle charge upon dissolution in aqueous medium) with the application. Accordingly, we can speak of non-ionic and ionic dyes; the latter being cationic and anionic in nature. They are classified according to the method of application as reactive, direct and acid (anionic dyes), basic (cationic dyes), or disperse and vat (non-ionic dyes) [20,38].

#### 1.3.1. Reactive Dyes

Reactive dyes make it possible to obtain a high wet strength compared to the less expensive direct dyes. However, their use is not always possible because of the difficulty in obtaining good unison. Another characteristic is that the chlorine-fastness is slightly lower than that of vat dyes, as is its light fastness under extreme conditions [39]. It has been reported that the reactive dyes are the only textile colorants that form a covalent bond with the substrate/textile fiber, usually cotton, during the application process under the influence of alkaline pH and heat [5,40,41]. Reactive dyes contain reactive groups such as vinyl-sulfone, chlorotriazine, trichloro pyrimidine, and difluoro-chloro pyrimidine, that covalently bond with the fiber during the dyeing process [42,43]. Adsorption results show that since reactive dyes are soluble in aqueous medium and have a greater negative charge density, the adsorption process was related to electrical attraction between anionic dyes and positively charged surfaces of adsorbent [43,44]. Initially, these dyes were designed for cellulose fibers, but nowadays they are used for cotton, wool and poly-amide fabrics; moreover some fiber-reactive dyes for protein and polyamide fibers are also commercially available [45]. With about 1150 entries in Color Index and ever rising volumes, the importance of reactive dyes in the global coloration business cannot be overemphasized. An equally well-known entrenched position is enjoyed by the chlorotriazines and vinyl-sulphones in the reactive system space, despite the introduction of at least one new reactive group every year from 1956 until 1971, except 1969 [46,47]. It is estimated that losses of 1–2% occur during the manufacturing process of dyes, while up to 1–10% of dyes are released back into the environment during use. For reactive dyes, the estimated loss is around 4%. [32,48]. According to other sources after the colorization process, approximately 10–50% of the initial dye load remains unused [49,50,51]. Reactive dyes are said to be the most problematic among other dyes, as they tend to pass through conventional treatment systems unaffected, therefore their removal is a difficult task [44,52].

#### 1.3.2. Direct Dyes

Direct dye is still the most widely applied in the dying and printing processes of the textile industry [53]. Direct dyes are water-soluble and anionic in nature, and they contribute 17% share in the textile industry, having wide utility in printing and dyeing cotton, viscose, silk, wool and leather [54,55,56]. Although these dyes are water-soluble anionic dyes, they cannot be classified as acid dyes because the acid groups are not the means of attachment to the fiber. Since these dyes do not require any kind of fixing, they are called direct dyes [45]. The major chromophore types are as follows: azo, stilbene, phthalocyanine, dioxazine, formazan, anthraquinone, quinolone and thiazole. Direct dyes are known to be easy to use, with a wide range of colors and shades, but have a low resistance to washing; this is what drives them out of the market compared to reactive dyes [39,57,58].

#### 1.3.3. Acid Dyes

Acid dyes, as their name implies, contain one or more acidic functions (SO_3_H and COOH) in their molecules [16]. They have excellent chemical and photochemical stability, which is why their industrial effluents have a complex composition, poor biodegradability and high tinctorial value [59,60,61]. This makes them difficult to remove by conventional methods. Their degradation products or metabolites can be potentially mutagenic or carcinogenic and can damage aquatic ecosystems. The use of water-soluble acid dyes, in particular sulphonic acid dyes, is very widespread due to their bright color and high solubility [16,60,62]. Acid dyes account for about 30% to 40% of total dye consumption. They are used in textiles, printing and dyeing, paper, leather, food, cosmetics, pharmaceutical and other industries for dyeing, e.g., nylon, wool, silk and modified acrylic [16]. The dye molecules are structurally very different and often contain some metal complexes. The defining characteristic of the group is the presence of sulphonated groups, which ensure water solubility, and azo-chromophore systems (the most important group), anthraquinone, triphenylmethane or copper phthalocyanine [39,43,45].

#### 1.3.4. Cationic-Basic Dyes

Basic dyes belong to the group of cationic dyes because they form a colored cationic salt in aqueous solution. Later, these cationic salts react with the anionic surface of the substrate (acrylic, paper and nylon). The resulting cations are electrostatically attracted to the negatively charged substrates [63].

The cationic functional groups (-NR^3+^ or =NR^2+^) are usually acid-soluble amino and substituted amino compounds. They would bind to the fiber by forming ionic bonds with its anionic groups [45].

In a literature study, it is recorded that this class of dyes is readily visible even at very low concentrations. This property contributes to the reduced efficiency of natural biological self-cleansing by blocking the penetration of sunlight, thus reducing photosynthetic activity. Basic dyes are highly resistant to degradation due to the number of aromatic rings associated with their resonance capacity, and their complex and large structure, which makes them durable and stable in the environment [64,65,66,67].

#### 1.3.5. Disperse Dyes

Disperse dyes are water-insoluble dyes; their structure is small and non-ionic with attached polar functional groups, such as -NO_2_ and -CN. They are applied to hydrophobic fibers from an aqueous dispersion [45]. They are mainly used for the dyeing of polyesters because they can interact with the polyester chains by forming dispersed particles. Disperse dyes are employed on cellulose acetate, nylon, acrylic fibers and cellulose fibers. The main classes are benzodifuranone, nitro, styryl, azo and anthraquinone groups [68]. Disperse dyes have a low solubility in water, therefore they must be applied with a dispersing aid, and are mainly used for acetate or polyester fiber [69]. From a chemical point of view, more than 50% of disperse dyes are simple azo compounds, about 25% are anthraquinones, and the rest are methine, nitro or naphthoquinone dyes [70]. Disperse dyes are also described as “sublimation” inks, as the ink molecules “sublimate” or change directly from solid to gas due to the application of heat, skipping any liquid state entirely [71]. The majority of disperse dyes are based on azo structures; however, violet and blue colors are often obtained from anthraquinone derivatives [16,72,73]. Disperse dye particles, due to their nano size, can keep better stability, especially in high temperature dyeing processes [74].

#### 1.3.6. Vat Dyes

Vat dyes are the main sources of pollution in the wastewater of textile and other industrial effluents, and they are widely used in dyeing cellulosic cotton fabrics [75]. These types of dyes are water-insoluble. Their main application is for cellulosic fiber, notably cotton dying [43]. Vat dyes are characterized by excellent color fastness, washability and chlorine-bleachable colored fibers [16,76]. The disadvantage of their application is that, as they are practically insoluble in water and thus have no affinity for cellulosic fibers, they are difficult to use (reduction and oxidation mechanisms) [77]. In conventional tank dyeing processes, the dye is reduced in alkaline medium with strong reducing agents, from which the most important is sodium dithionite (Na_2_S_2_O_4_) [78,79].

Nirav P. Raval et al. [45] made a detailed classification in their article, where the dyes are grouped based on:

- source of materials/origin (natural–substantive and adjective–synthetic);
- method of application to the substrate (acid, basic, direct, mordant, reactive, disperse, solvent, sulfur);
- their chemical structure (azo, nitro, indigoid, cyanine, xanthene, quinione-imine, acridine, oxazine, anthraquinone, phthalein, triphenylmethane, nitroso, diarylmethane); and
- the electronic origins of color (donor–acceptor chromogens, polyene chromogens, n→π^2^ chromogens, cyanine type chromogens) [16,43,45].

## 2. Dye Removing Methods, Technologies

Dye removal methods have been summarized in review articles by many authors [5,80,81,82,83,84,85,86,87,88,89,90,91,92,93]. The importance of removing dyes is driven by a number of factors; they are harmful to health, often mutagenic and carcinogenic, inhibit photosynthetic activity in the aqueous medium, and even at very low levels (<1 ppm) are highly visible and undesirable in water bodies, with color being the most obvious parameter affecting water quality [94,95]. Hessel C. et al. described the percentage of non-fixed dye that may be discharged in the effluent as a function of dye classes from EPA and OECD legislation [5].

Throughout recent years, numerous investigations have been made to find the ideal technology for dye wastewater purification. Even though a high range of methods have been studied in the past 30 years, only several are truly being implemented by the concerning industries these days due to the limitations they possess [5].

**Figure 3:**
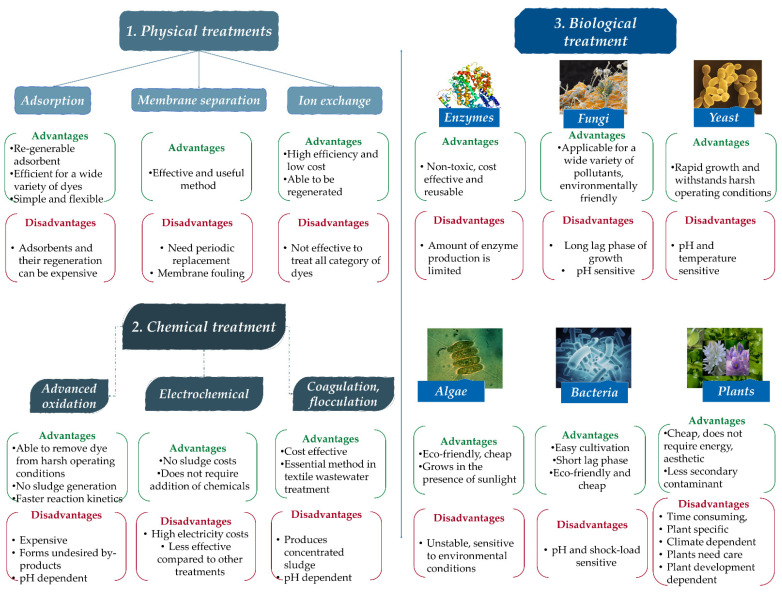
Dye removing methods and their advantages/disadvantages [89].

As it appears in the review articles referred to above, dye remediation technologies can be divided into three main categories: physical, chemical, and biological methods. As a summary, Figure 3 contains some of the used methods, and their advantages and disadvantages [89].

Review articles exclusively analyze and compare paint removal methods. Often, published studies are used to illustrate the effectiveness of the methods presented. In these studies, several methods are classified into the three main categories of paint removal [5,82,83,87,89]. Physical dye removing techniques can be: adsorption, membrane separation, reverse osmosis, ion exchange, ultrasonic mineralization, nano-remediation and photo-Fenton processes. Chemical methods are: catalytic reduction, coagulation/flocculation, electrochemical reduction, photolysis/photochemical reduction, advance oxidation processes, ultraviolet irradiation ozonation, clay minerals and zeolites. Biological methods can be divided to phytoremediation and microbial remediation (bacterial, algae, fungi, mycoremediation, enzyme degradation and phycoremediation) [5,37,96].

## 3. General Aspects of Adsorption Process

The term adsorption was first used in 1881 by the German physicist Heinrich Kayser [97]. The past decade has seen a boom in environmental adsorption studies on the adsorptive removal of pollutants from the aqueous phase. It is preferred over other methods because of its relatively simple design, operation, cost effectiveness, and energy efficiency [98].

It is a mass transfer process in which a substance (adsorbate) moves from a gas or liquid phase to form a surface monomolecular layer on a solid or liquid condensed phase (substrate, the adsorbent). It usually involves the molecules, atoms or even ions of a gas, liquid or solid in a dissolved state that are attached to the surface. In practice, adsorption is performed as an operation, either in batch or continuous mode, in a column packed with porous sorbents [99].

Adsorption is often confused by the term absorption. The difference between absorption and adsorption is that in absorption the molecules penetrate a three-dimensional matrix, while in adsorption the molecules attach to a two-dimensional matrix [100,101,102]. The process is usually reversible (the reverse process is called desorption), so that sorption is responsible not only for the extraction of substances but also for their release.

**Figure 4:**
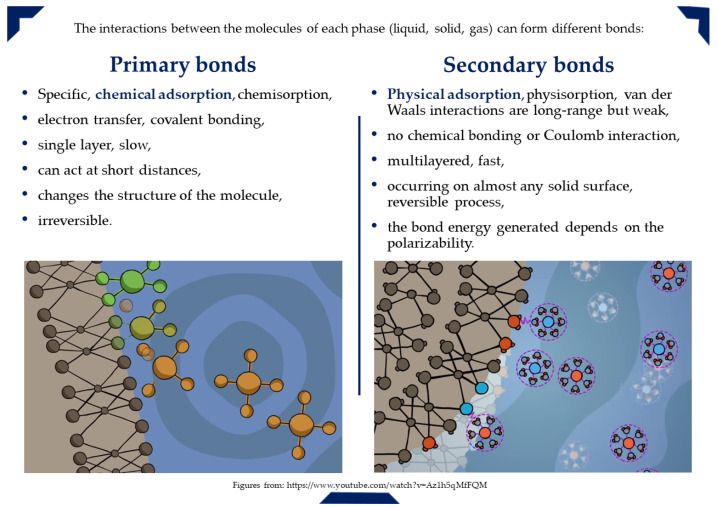
Types of adsorption bonds and nature of adsorption.

Adsorption can occur due to physical forces or chemical bonds, primarily as a result of surface energy. In general, partially exposed surface particles tend to attract other particles into position. There are several ways of classifying adsorption, and Figure 4 provides a classification based on the nature of the bond (physical or chemical bonds) formed between the adsorbent and the pollutant, describing its characteristics [103,104,105].

Since adsorption phenomena occur in many natural, biological, physical and chemical systems, people tend to apply it in industrial processes and take advantage of its benefits. It is increasingly used for purification or separation purposes; it is also a wastewater treatment technique for the removal of a wide range of compounds from industrial wastewater due its low cost and easy operation [102,106]. Adsorption is most commonly performed to remove low concentrations of non-degradable organic compounds from groundwater, drinking water production, process water, or as tertiary treatment, for example after biological water purification [107].

In summary, adsorption, surface enrichment, refers to the binding of atoms, ions and molecules on the active centers of a solid surface (surface binding).

In most cases, the method does not require unnecessary energy input; the removal rate often depends on the kinetic equilibrium and is determined by the surface characteristics and composition of the adsorbent. The progress of adsorption depends largely on the affinity of the adsorbent, its ability to react with the pollutant and the adsorption mechanism between the sorbent and the functional groups of the pollutant [108,109,110]. The end point of the adsorption process is considered to be the concentration value at which equilibrium stability between the solid and liquid phase volumes is reached [110].

### Possible Adsorbents

A wide range of review articles [93,111,112,113,114,115,116,117,118,119,120,121,122] discuss the use, classification, effectiveness and properties of different adsorbents as they are some of the key influencing factors of the process. The characteristics through the advantages and limitations of most adsorbents are also reviewed. This is due to the fact that in recent years, researchers have focused their attention on the use of new, alternative, cost-effective, environmentally friendly, green adsorbents to replace the commonly used activated carbon [86]. Since adsorption processes are required to have high removal efficiency even at trace levels, it is crucial to investigate and develop new adsorbents with better properties, i.e., low cost and easily accessible. The adsorbents may be collected from agricultural or animal waste, or industrial by-products. All adsorbents, by their intrinsic nature, have functional groups that play the key role in adsorption; therefore, the type of the adsorbent is a key factor in the waste removal process [123].

Each adsorbent has its own characteristics, such as porosity, pore structure, adsorbent surface area, and structural specificity [124]. A high range of adsorbents have been studied to remediate dye contaminated waters: clays [125,126,127], chitosan [128,129], cyclodextrin [130,131,132], eggshell [51,133,134,135], orange peel [136], fluorene-based covalent triazine framework [137], cellulose [138], wool [139], shrimp [140], rice bran hydrogel beads [141], coccine [142], seeds [143,144].

With the increase in the number of adsorbents used, their classification and sorting has become indispensable. The different types of adsorbents can be classified in several ways; however, the most common ones are listed below [145]:

- natural materials: sawdust, wood, fuller’s earth or bauxite;
- natural materials treated to develop their structures and properties: activated carbons, activated alumina or silica gel;
- manufactured materials: polymeric resins, zeolites or alumino-silicates;
- agricultural solid wastes and industrial by-products: date pits, fly ash or red mud;
- biosorbents: chitosan, fungi or bacterial biomass.

Another classification is based on their origin:

- *Natural adsorbents* include carbon, clays, clays minerals, zeolites and ores. These natural materials are often relatively inexpensive, abundant, plentiful and readily available;
- *Synthetic adsorbents* are adsorbents produced from agricultural products and wastes, household wastes, industrial wastes, sewage sludges and polymer adsorbents.

We can distinguish five main categories of novel adsorbents [86]: (i) clay/zeolites and composites; (ii) biosorbents; (iii) agricultural solid wastes; (iv) industrial by-products and their composites; (v) miscellaneous adsorbents. Biosorbents further include chitosan, cyclodextrin, biomass and their composites. Agricultural solid wastes, as adsorbents, include sawdust, bark and other materials like cotton fiber, coffee/tea residues, rice husk, different vegetable and fruit peels and their composites. The industrial by-products include metal hydroxide sludge, fly ash and red mud. Nanomaterials and metal organic frameworks are examples of miscellaneous adsorbents.

Requirements for sorbents [112]:

- Ability to work under several wastewater parameters;
- Cost effectiveness;
- Removal capability of diverse contaminants;
- High adsorption capacity;
- High selectivity for various concentrations;
- High porosity and specific surface area;
- High durability;
- Reusability of adsorbent, ease of regeneration;
- Fast kinetics; and
- Being present in large quantities.

## 4. Factors Affecting Adsorption Process

**Figure 5:**
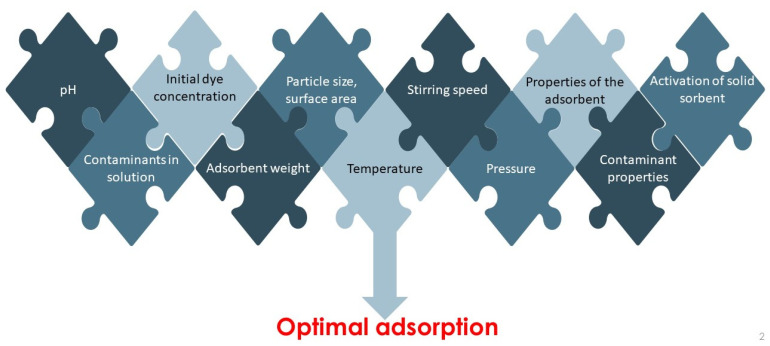
Factors affecting adsorption process.

The efficiency of liquid phase adsorption, and therefore the optimal operation of the water treatment process, depends on several parameters. The sorption performance, as illustrated in Figure 5, is influenced by physico-chemical factors, the type of pollutant (in this study, the dyes) and its chemical structure, and the properties of the adsorbent used. Such physicochemical parameters are the adsorbent/adsorptive interaction, the surface chemistry and pore structure of the adsorbent, particle size, nature of the adsorbent, presence of other ions in the aqueous solution, pH, temperature, pressure, and contact time. The properties of the adsorbate, its molecular weight, molecular structure, molecular size and polarity should also be taken into account [38,146].

In a batch process, the mixing speed of the aqueous suspension may affect the time required to remove the contaminant. When a solid sample of known mass is exposed to a liquid phase of known composition, the concentration varies continuously until equilibrium is reached as a result of the multiplication. The time required for this, which can be effectively reduced by shaking or stirring, is determined from preliminary kinetic measurements. The amount adsorbed can be calculated from the initial and equilibrium composition and the amount of the materials (solid mass and liquid volume). The rate is also experiment-dependent (adsorbent, contaminant, adsorption method). In general, increasing the rate will increase the biosorption removal rate of adsorbed impurities by minimizing mass transfer resistance, but may damage the physical structure of the biosorbent [147,148,149,150,151,152].

In contrast to most laboratory experiments, the effluent of industrial water treatment is not only a single component. Industrial wastewater contaminated with dyes can contain a number of hazardous chemicals: acetic acid, ammonium sulphate, caustic soda, dispersing agent, formic acid, hydrochloric acid, hydrogen peroxide, hydrosulphates, leveling agent, organic resign, organic solvent, oxalic acid, polyethylene emulsion, PV acetate, soap, softener, sulfuric acid, and wetting agent [5]. A wide range of contaminants occur in wastewater, such as heavy metals, pesticides, pharmaceutical residues, dyes and colloidal particles. These can all affect adsorption removal through competition for binding sites or other interferences. Increasing concentrations of competing contaminants tend to reduce biosorption removal of the target contaminant [153].

The effects of all these parameters should be taken into account when designing an adsorption process. Optimization of such conditions will greatly aid the development of industrial-scale dye removal technology. The most studied influencing factors (initial dye concentration, aqueous solution pH, adsorbent volume and particle size, and temperature) are illustrated with the results of research over the last five years. General trends are formulated based on the results obtained, considering the effects of the factors.

### 4.1. The Effect of Initial Dye Concentration

The initial dye concentration is perhaps one of the most important factors influencing the adsorption process, as it indirectly affects the efficiency of dye removal by reducing or increasing the availability of binding sites on the adsorbent surface. In such water treatment systems, the efficiency of dye removal (E) and the maximum amount of dye bound in equilibrium (q) are directly related to the initial dye concentration [38,154].

(1) $$E\left(\%\right)=\frac{C_{i}-C_{f}}{C_{i}}\cdot 100$$

(2) $$q=\frac{\left(C_{i}-C_{f}\right)\cdot V}{m}\;\;\;$$

where: E (%)—efficiency; q (mg/g)—amount of dye bound in equilibrium; C_i_ (mg/L)—initial dye concentration; C_f_ (mg/L)—final dye concentration; m (g)—amount of adsorbent; and V (L)—volume of aqueous solution.

By examining the effect of initial dye concentration, three trends can be observed (exemplified in Table 1):

- the removal efficiency decreases as the initial concentration increases;
- removal efficiency increases as the initial concentration increases; and
- no significant change in removal efficiency.

**Table 1:** Results of various research regarding the effect of initial dye concentration.

Dyestuff	Adsorbent	Concentration (mg/L)	Reaction Time (min)	Efficiency Range (%)	Quantity in Equilibrium Range (q_e_ mg/g)	Reference
Methylene Blue	Algerian palygorskite	3–30	5	up to 97%	2.5–10	[155]
Methylene Blue	clinoptilolite	50–100	60	increased but no significant difference > 95%	-	[156]
Brilliant Green	activated carbon derived from medlar nucleus	110–200	60	-	100–180	[157]
Methylene Blue	green olive stone	50–1000	24 h	fluctuating values, highest 65.9 at 50 ppm	-	[158]
Methylene Blue	black olive stone	50–1000	24 h	fluctuating values, highest 93.5 at 400 ppm	-	[158]
Acid Brown	*Haloxylon recurvum* plant	10–60	180	-	2.846–10.011	[159]
Congo Red	cocoa bean shells	40–120	4–36 h	negative linear effect		[160]
Methylene Blue	fava bean peels, utilizing ultrasonic-assisted (US) shaking	3.6–100	70	70–90	-	[161]
Methylene Blue	fava bean peels, conventional (CV) shaking	3.6–100	70	80–95	-	[161]
Reactive Blue 19	corn silk	10–500	60	-	2.0–71.6	[162]
Reactive Red 218	corn silk	10–500	60	-	2.0–63.3	[162]
Reactive Black 5	pent tea leaves	50–100	5–200	98.7–43.5	24.8 –6.7	[163]
Methyl Orange	pent tea leaves	50–100	5–200	88.7–32.7	22.2 –1.6	[163]
Methylene Blue	*Citrus limetta* peel	5–25	10–60	~100–97	0.06–1.62	[164]
Malachite Green	*Citrus limetta* peel	5–25	10–60	~97–95	0.17–4.70	[164]
Congo Red	*Citrus limetta* peel	5–25	10–60	~90–75	0.17–3.77	[164]
Crystal Violet	mango stone biocomposite	20–50	60	-	~25–352.79	[165]
Congo Red	chitosan	50–2000	30	-	increased to 0.2	[166]
Methylene Blue	chitosan	25–100	30	~100–50	increased to 1457.1	[166]
Rhodamine B	chitosan	25–100	30	~55–35	increased to 990	[166]
Reactive Red 120	*Moringa oleifera* seed	10–100	30	-	18.54–173.99	[167]
Crystal Violet	olive leaves powder	10–100	5–70	-	~5–45	[168]

Most often, the percentage of dye removal decreases with increasing initial paint concentration. This phenomenon can be explained by the saturation of adsorption sites on the adsorbent surface. In this case, as the initial concentration increases, so does the capacity of the adsorbent, which is due to the high mass transfer driving force at high initial dye concentrations. The initial concentration of solute acts as a driving force for the adsorption process, favoring diffusion and mass transfer processes from the solution (with a higher amount of dye) to the free surface of the adsorbent [158,169].

If the concentration of the solution increases, and with it, the amount of bound material shows a similar trend, then at low initial solution concentration the surface area of the adsorbent and thus the number of adsorption binding sites is high, so the contaminant ions or molecules (in our case dye molecules) can easily bind to the adsorbent surface. At higher initial solution concentrations, the total available adsorption sites are limited, which may result in a reduction in the percentage removal of contaminants. The increase at higher initial concentrations may be attributed to increased driving forces [170,171].

At low concentrations, the ratio of active sites to dye molecules can be high, allowing all molecules to interact with the adsorbent and be removed from solution almost instantaneously [172].

Arellano G. Rodríguez et al. [160] reported that a negative linear effect between removal efficiency, amount of bound material and initial concentration occurred when removing Congo red with cocoa bean shells [160]. Accordingly, as the initial dye concentration increased, the adsorption capacity of the biosorbent decreased. Referring to other similar studies with Congo red, it was explained that the equilibrium adsorption capacity increases with increasing initial dye concentration, a process controlled by the mechanism of resistance to removal of Congo red [160].

Even though it is a driving force, a clear, generalizable influence of the initial concentration as a parameter is not possible since several experimental conditions act in combination on the specific contaminant and the adsorbent under study.

### 4.2. The Effect of Solution pH

According to several papers, the key parameter in almost all adsorption processes is the pH of the dye solution. This factor affects the capacity of the adsorbent and the efficiency of the process.

The pH affects the solution chemistry of contaminants, the activity of functional groups in the adsorbent, the competition with coexisting ions in the solution, and the surface charge of the adsorbent. The pH of the aqueous medium can also influence the properties of the adsorbent, the adsorption mechanism, and the dissociation of dye molecules. Not only the adsorbent but also the chemical structure of the dye can be altered by the pH of the solution. The pH changes the surface charge and the degree of ionization of the adsorbed ion [133,173,174,175,176,177].

Practical applications (Table 2) demonstrate that anionic dyes bind more effectively to the adsorbent surface in acidic media, whereas cationic dyes bind more effectively in basic media. Usually, the pH of the aqueous dye solution is adjusted with HCl and NaOH.

- When HCl is added to the solution, the surface of the adsorbent in the solution is protonated, allowing the anionic dye to bind more efficiently on its surface, due to the electrostatic attraction.
- Conversely, in basic medium, the addition of NaOH deprotonates the biomass surface, resulting in a repulsive force between the anionic dye and the biomass. Thus, the reverse phenomenon is observed for cationic dyes.

**Table 2:** Results of various research regarding the effect of initial solution pH, where E is the efficiency of the adsorption process and E_max_ is the highest efficiency calculated in the specific article at a given condition.

Dyestuff	Adsorbent	Dyes Ionic Nature	pH	Observations: with the Increase (↑) of pH	Reference
Direct Red 5B	spent mushroom waste	anionic	2 to 10	E% ↓; E_max_pH=2_ = 95%	[178]
Direct Black 22		anionic	2 to 10	E% ↓; E_max_pH=2_ = 98%	[178]
Direct Black 71		anionic	2 to 10	E% ↓; E_max_pH=2_ = 95%	[178]
Reactive Black 5		anionic	2 to 10	E% ↓; E_max_pH=2_ = 96%	[178]
Congo Red	powdered activated carbon: rubber seed	anionic	4 to 11	E% ↓	[179]
	powdered activated carbon: rubber seed shells		4 to 11	E% ↓	[179]
Methylene Blue	powdered activated carbon: rubber seed	cationic	4 to 11	E% ↑	[179]
	powdered activated carbon: rubber seed shells		4 to 11	E% ↑	[179]
Eriochrome Black T	powdered vegetables wastes	anionic	2 to 10	E% ↓; 50.65 to 4.01%	[180]
	calcined vegetables wastes		2 to 10	E% ↓; 68.87 to 31.23%	[180]
Methyl Orange	natural olive stone	anionic	2 to 12	q (mg/g) ↓; 26.4 to 3.3 mg/g	[158]
	olive stone activated carbons		2 to 12	q (mg/g) ↓; 120 to 15 mg/g	[158]
Methylene Blue	natural olive stone	cationic	2 to 12	q (mg/g) ↑; 18 to 120 mg/g	[158]
	olive stone activated carbons		2 to 12	q (mg/g) ↑	[158]
Reactive Orange 16	carbon from *Phyllanthus reticulatus*	anionic	2 to 11	q (mg/g) ↓	[181]
Cationic Red X-5GN	ceramic	cationic	2 to 10	E% ↑	[182]
Cationic Blue X-GRRL		cationic	2 to 10	E% ↑	[182]
Methylene Blue	activated carbon/cellulose biocomposite films	cationic	3 to 11	q (mg/g) ↑; 50.54 to 60.48 mg/g	[183]
Eriochrome Black T	almond shell	anionic	2 to 11	q (mg/g) ↓	[184]
Malachite Green		cationic	2 to 11	q (mg/g) ↑	[184]
Basic Yellow 37	bast fibers: ramie	cationic	2 to 12	E% ↑; E_max_pH=12_ = 91%	[185]
	bast fibers: flax	cationic	2 to 12	E% ↑; E_max_pH=12_ = 88%	[185]
	bast fibers: kenaf	cationic	2 to 12	E% ↑; E_max_pH=12_ = 78%	[185]
Remazol Brilliant Violet	*Trichoderma viride*	anionic	4 to 9	E% ↓; 79.05 to 50.25%	[186]
Congo Red	eggshell powder	anionic	2 to 10	E% ↓; 98.71 to 93.17%	[133]
Bromphenol Blue		anionic	2 to 10	E% ↓; 67.61 to 1.2%	[133]
Methylene Blue		cationic	2 to 10	E% ↑; 14.8 to 75.1%	[133]
Malachite Green		cationic	2 to 10	E% ↑; 89.95 to 97.92%	[133]

### 4.3. The Effect of Adsorbent Dosage

The amount of adsorbent is an important parameter that influences the adsorption process, through the quantitative ratio of adsorbent to adsorbent. Since the adsorbent determines the adsorbent capacity for a given initial concentration, the dosage of the adsorbent is an important parameter [187]. According to Kroeker’s rule, the specific adsorbed volume, for a constant initial concentration, decreases with increasing adsorbent mass [188]. Thus, increasing the adsorbent dose is positively correlated with the efficiency and performance of dye removal. With increasing adsorbent dosage, at fixed contaminant concentrations, more active surface area is available for adsorption and more active adsorption sites are available [189].

As the concentration of biomass (the amount of adsorbent) increases, the efficiency of pollutant removal (E%) increases, but there is no direct proportionality between the amount of biomass and the amount of pollutant removed (q_e_).

In contrast, as the concentration of biosorbent increases, the amount adsorbed per species (q_e_) decreases. This can be attributed to the fact that the shape of the sorption isotherm changes with increasing biosorbent concentration. The decrease in the specific adsorbed amount is probably due to the fact that some of the surface or surface groups may not be saturated in the more concentrated suspensions [190,191,192,193].

During the dye removal process, the capacity may decrease for two reasons [194]:

- adsorption sites remain unsaturated while the number of sites available for adsorption increases; or
- aggregation or agglomeration of adsorbent particles may occur, reducing the available surface area and increasing the diffusion path length.

Scientific studies in recent years have investigated the removal of different dyes with different amounts of broad-spectrum adsorbent. Some examples of these are listed in Table 3 to support the detected relationships between mass and adsorption. It is observed that at fixed pollutant concentrations, as the mass of the adsorbent increases, the efficiency increases, and the maximum amount of material bound decreases.

Several studies also report that this increase in efficiency lasts until a saturation state is reached and then steadily decreases, sometimes slightly. This can be explained by the fact that after a certain adsorbent dose, maximum adsorption is reached and the amount of ions bound to the adsorbent and the amount of free ions remains constant, even with the further addition of adsorbent [51,134,135,187,192,197].

### 4.4. The Effect of Adsorbent Particle Size

Although not regularly investigated in biosorption studies, particle size can be an important factor in heterogeneous chemical reactions and adsorption [203]. The small particle sizes result in a higher specific surface area. Specific surface area (SSA), defined as the total surface area of a solid material per unit of mass, is an important feature for sorption processes. SSA is dependent on the size of the particles, as well as on the structure and porosity of the material [204]. The most common unit of measurement is m^2^/g.

The relationship of adsorption capacity to particle size depends on two criteria [205,206]:

- the chemical structure of the dye molecule (its ionic charge) and its chemistry (its ability to form hydrolyzed species); and
- the intrinsic characteristic of the adsorbent (its crystallinity, porosity and rigidity of the polymeric chains).

In adsorption by static batch methods, smaller particle sizes result in higher adsorption capacity and efficiency, since there are more active sites for binding [207]. Table 4 represents results of studies where the effect of particle size was investigated, and a similar trend was observed. With the decrease of the particle size, the BET surface of the material increases.

**Figure 6:**
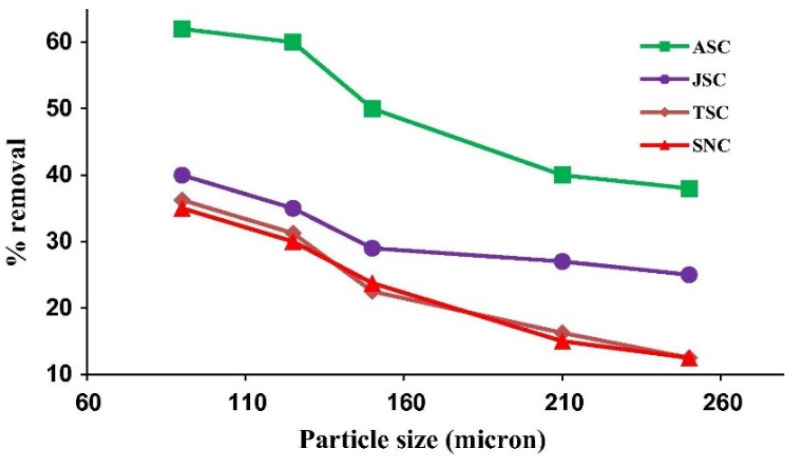
Representation of particle size trends, where the used adsorbents are ASC—aamla seed carbon, JSC—jambul seed carbon, TSC—tamarind seed carbon, and SNC—soapnut carbon [213].

Figure 6, from the study of Shahul K. Hameed et al. [213], represents the effect of particle size on adsorption efficiency, where chromotrope dye was adsorbed on the surface of activated carbons obtained from the seeds of various plants.

If the particle size is too small, the adsorption capacity may be lower, depending on the type of adsorbent, as the lighter particles float and thus cannot contact the solution. The separation of these small particles from water after biosorption can be challenging [203].

### 4.5. The Effect of Solution Temperature

The effect of temperature is also a significant physico-chemical factor as it affects the treatment process by shifting the nature of the reaction from endothermic to exothermic, or vice versa [9]. Moreover, it has a strong effect on the adsorption as it can increase or decrease the amount of adsorption [214].

The temperature can affect the efficiency of the sorption differently depending on the adsorbent and the pollutant. In general, it enhances biosorption of adsorption impurities by increasing the surface activity and kinetic energy of the adsorbate, but it can also damage the physical structure of the biosorbent.

- As the temperature rises, the rate of chemical reaction also increases, so if the sorption process is chemisorption (∆H_chemisorption_ = −200 kJ/mol), then higher sorption efficiency will be seen at higher temperatures (this would eventually reach equilibrium).
- On the other hand, if the process is a physical adsorption (∆H_physisorption_ ≈ −20 kJ/mol), then the higher temperature will negatively affect the adsorption. Temperature can chemically alter the adsorbent, its adsorption sites and activity [110].

We can differentiate two types of processes: endothermic and exothermic (Table 5).

Exotherm: with the increase of temperature, the adsorption process (efficiency) decreases. It can be explained with the fact that the adsorptive powers among adsorbate and the active sites of the adsorbent become weak with the increase in temperature, and dye removal efficiency decreased [215]. Exothermic adsorption is usually used to control the diffusion process, as the mobility of the dye ions increases when heat is added to the system [216].

Endotherm: with the increase of temperature, the adsorption process (efficiency) increases, due to more availability of active sites as a result of the activation of the adsorbent surface at higher temperatures [217]. Increasing the values of adsorption capacity by increasing the temperature may be attributed to an increase in the mobility of the large dye ions [218].

All in all, better adsorption at higher temperatures may indicate the endothermic nature of the process, while being exothermic at lower temperatures.

**Table 5:** Results of various research regarding the effect of temperature.

Dyestuff	Adsorbent	Temperature (K)	Efficiency Range (%)	Type of the Process	Quantity in Equilibrium Range (q_e_ mg/g)	Reference
Basic Orange 2	alkaline-modified nanoclay	288–308	80–100	endothermic	-	[219]
Congo Red	cross-linked TTU-chitosan	298, 308 and 328	-	endothermic	increased	[218]
Congo Red	modified Zeolite A	297–309	-	exothermic	decreased	[216]
Direct Sky Blue	ZnO	Beyond 313 K, the adsorption capacity was decreased, which is an indication of being endothermic up to 313 K, and exothermic beyond this temperature			highest: 40.94	[220]
	MgO				highest: 46.25	[220]
	FeO				highest: 42.86	[220]
Methyl Orange	cationic polymer (Amberlite IRA 402)	293, 303, 328 and 348	-	endothermic	increased	[221]
Remazol Red	chitosan Schiff base	293, 303, and 313	-	endothermic	increased	[222]
Reactive Red 120	activated carbon	The adsorption of RR-120 on activated carbon is of the physisorption type, as confirmed by the adsorbed energy values, and it is exothermic as verified by the internal energy				[223]
Methylene Blue	hydroxyapatite/gold nanocomposite	290–305	-	endothermic	increased	[217]
		305–330	-	exothermic	decreased	[217]
Reactive Red 35	multiwalled carbon nanotubes	298, 308, 318 and 328	63.33–9.07	exothermic	-	[215]
	poly (acrylonitrile-styrene) impregnated with activated carbon	298, 308, 318 and 328	67.55–97.61	endothermic	-	[215]
Methylene Blue	*Citrullus colocynthis* seed	293–333	93.58–98.00	endothermic	-	[143]
	*Citrullus colocynthis* peel	294–333	91.43–82.52	exothermic	-	[143]
Methylene Blue	magnetic carboxyl functional nanoporous polymer	298, 308 and 318	-	endothermic	52.16–52.58–53.75	[224]

After studying the effect of initial temperature on adsorption, thermodynamic parameters are calculated. It is well known that the adsorption processes are strongly dependent on the working temperature, which is controlled by thermodynamic parameters including the standard enthalpy change (ΔH_0_, J/mol), the standard entropy change (ΔS_0_, J/mol) and the standard free Gibbs energy change (ΔG_0_, J/mol) of the adsorption processes. These parameters are computed from the Gibbs–Helmholtz equation: ΔG = ΔH − TΔS [225]. Gibbs free energy, enthalpy and entropy are state functions, so ΔG, ΔH and ΔS depend on the final state and the initial state of the adsorption system. Gibbs free energy, enthalpy and entropy have extensive property, so attention must be paid to the amount of substance that these thermodynamic parameters correspond to [226].

During the adsorption of dye molecules, with the increase of temperature, the value of entropy (ΔS) and enthalpy (ΔH) can be increased or decreased.

Molecules before adsorption can move in three dimensions, but as they get adsorbed on the surface, the motion of them is restricted towards the surface, and their disorder decreases, resulting in the decline in entropy indicating an exothermic process. This may also be explained on the basis that the solubility of dyes increased at higher temperatures while adsorbate–adsorbent interactions decreased, resulting in decreased adsorption [227]. The increase in entropy and enthalpy indicates an endothermic process [225,228,229,230,231].

### 4.6. Activation of Solid Sorbent, Surface Modification

In order to increase the adsorption capacity and efficiency, different types of physical and chemical surface modification methods can be used. The most common physical modification methods are freezing, crushing, boiling/heating and drying. These types of surface modification techniques usually destroy the cell membrane of the biomass, releasing cellular content that may be responsible for contaminant uptake.

Physical modification methods are generally cheap and simple, but not as effective as chemical methods. Among the chemical modification methods, polymerization, modification of the binding site, and washing (or pretreatment) are being experimented with. Of the chemical methods, washing is preferred for its simplicity and efficiency. The most common chemical pretreatments include washing of biomass with acid, alkali and detergent, or crosslinking with organic solvents. Some types of adsorbents produce stable biosorbent particles after some simple processes such as cutting or grinding. In other cases, the adsorbent must be fixed in a synthetic polymer matrix and/or grafted onto an inorganic carrier material such as silica in order to obtain particles with the required mechanical properties. Different ways of manipulating biomass adsorbents to improve various aspects of biosorption have been described by several authors [232,233,234,235,236,237].

## 5. Desorption Studies

Desorption studies help to explain adsorbate and adsorbent recovery, and the adsorption mechanism. Since the regeneration of the adsorbent makes the treatment process economical, desorption studies were performed to regenerate the spent adsorbent [187]. As batch adsorption is not a destructive technique and the adsorbents used undergo a phase transformation, large amounts of often hazardous by-products and waste are generated. These solids can be regenerated due to their properties, leaving room for the recovery of the adsorbent and often the contaminant [94]. The process of adsorbent regeneration is a complex task, as the desorption depends on the adsorbent, the adsorbate (different types of dyes ionic nature), and the adsorption process. In adsorption–desorption studies, it is essential to examine the reusability of the adsorbent. Between dye removals, the adsorbent should be cleaned and regenerated to ensure that it can continue to be used and the water treatment can be reproduced. The adsorbent lifetime expresses the number of adsorption–desorption cycles, after which the adsorbent can be used effectively to remove dye substances. Therefore, the task of scientists who study the desorption process is to provide information about the reproduction cycles. There are different desorption methods, and a high range of eluents are employed to regenerate the used adsorbents, out of which a few examples will be listed below.

The reuse of adsorbent could be considered as one of the most important economic parameters. Siroos S. et al. studied the recyclability of NaX nanozeolites after malachite green (MG) and auramine-O (AO) dye adsorption. The NaX nanozeolites used were washed with a small amount of methanol and then dried for reuse in a vacuum-oven. The results showed that after up to five cycles, the adsorption efficiency decreases slightly. In general, this reduction can be due to adsorption degradation during adsorption–desorption cycles [238].

Feng J. et al. examined the desorption of cationic malachite green (MG) dye on cellulose nanofibril aerogels. For this purpose, the used aerogels in the first round were put in deionized water, after the treatment 16% of MG was regenerated. Another desorption method consisted of putting the material in 50 mL of 0, 50 and 200 mM sodium chloride solutions. As a result, after 1 h, 65 (50 mM) and 85% (200 mM) recovery was observed [239].

Haq N.Bhatti et al. made a detailed research about the adsorption–desorption behavior of Direct Orange-26 (DO-26), Direct Red-31 (DR-31), Direct Blue-67 (DB-67) and Ever direct Orange-3GL (EDO-3) dyes onto native, modified rice husk. The dyes desorption was investigated using distilled H_2_O (pH 8, 10, 12), NaOH and Na_2_CO_3_ (0.1 M) after drying of the biosorbent at 60 °C. It was observed that the EDO-3, DR-31, DO-26 and DB-67 dye can be desorbed from rice husk biomass under basic conditions and 75.32, 80.59, 62.88 and 53.97 (mg/g) respectively. The adsorption capacity of rice husk biomass has lost 17% at the end of ten sorption/desorption cycles [240].

The adsorption–desorption of Acid Violet 17 was examined by İlknur Şentürk and Mazen Alzein regenerating acid-activated pistachio shell [187]. As a protocol, 1 g of the dye-loaded adsorbent obtained (0.1, 0.2, 0.4, 0.8 M) was mixed separately with 100 mL of HCl, NaCl, CH_3_COOH, NaOH desorption agents prepared at different concentrations (0.1, 0.2, 0.4, 0.8 M) and solvents (ethanol and distilled water) in the orbital mixer operating at 125 rpm for 24 and 48 h. The desorption efficiency was very low in desorption processes performed separately with water and ethanol. The AV 17 dye adsorption efficiency after three cycles of desorption decreased from 94.76 to 75.84% [187].

Mohammad A. Al-Ghouti and Rana S. Al-Absi made desorption studies where spent black and green olive stones loaded with 600 mg/L methylene blue were added to 50 mL of acidic mixtures of acetic acid and ethanol (%vol) (10:1, 5:1, and 1:1). The mixture was then shaken at 25°C and 150 rpm for 24 h. The total desorption removal capacities of the MB-loaded black and green olive stones were found to be 92.5 and 88.1%, respectively [158].

A chemical regeneration experiment was conducted by Momina et al. on the surface of bentonite after methylene blue dye adsorption. The used solvents were: hydrochloric acid (HCl), nitric acid (HNO_3_), ethanol (C_2_H_5_OH), propanol (C_3_H_7_OH), acetone ((CH_3_)_2_CO), sodium chloride (NaCl), sodium hydroxide (NaOH) and distilled water (H_2_O) [241]. Significant desorption of MB (70%) was achieved using aqueous HCl solution.

Direct Blue 78 adsorption–desorption on eggshell surface was analyzed using NaOH solvent by Ainoa Murcia-Salvador et al., where results showed that the adsorption abilities of the eggshell decreased with the increasing number of cycles [242].

**Figure 7:**
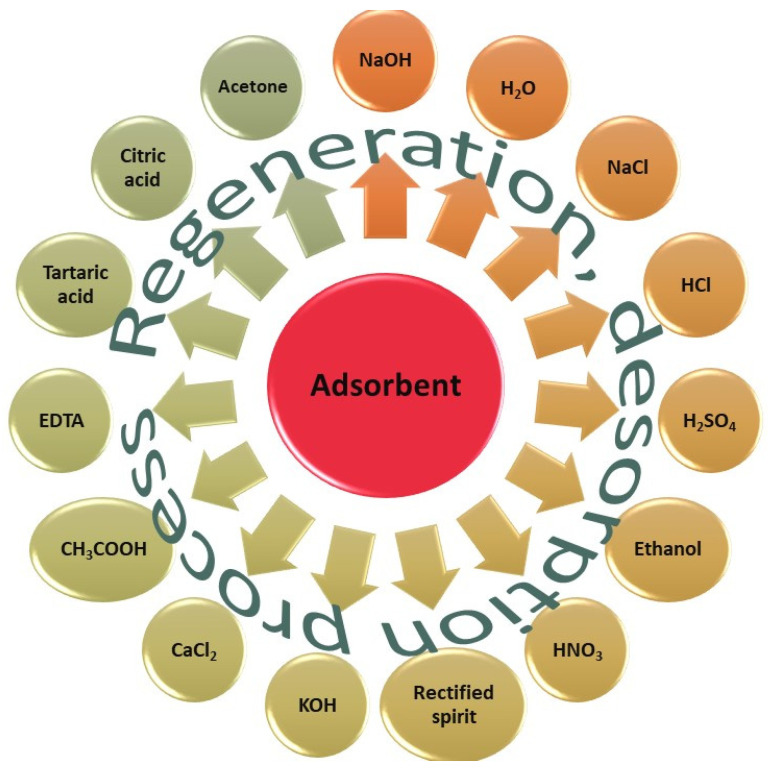
Possible eluents used to desorb contaminants from adsorbent materials.

Figure 7 contains possible eluents used to desorb contaminants from adsorbent materials; therefore, to regenerate them.

In the Journal of Saudi Chemical Society, Himanshu Patel wrote a review article about the comparison, advantages, and disadvantages of different adsorbent regeneration processes. Moreover, it lists a high range of eluents used by other researchers [243]. As he writes in the abstract of the article, hazardous solid waste is one of the most serious problem faced all over the World, which comprises spent solid adsorbents.

## 6. Conclusions

In the first chapter of the study, we discussed that since ancient times, people have used dyes to paint their everyday objects. As a result of population growth and a large increase in industrial production, increasing quantities of dyes were needed. With the development of science and the chemical industry, researchers have found a solution to this problem; they have developed various synthetic dyes, the large quantities of which required classification and catalogization, but have also created another issue that is harmful to the environment and health. We must therefore tackle the challenge of treating industrial wastewater (mainly dyes and textiles) and develop appropriate and sustainable water treatment technologies.

Several possible methods for water treatment have recently become available, but adsorption is perhaps the most common commercial treatment. The remediation process is influenced by several external parameters, the optimization of which is essential to ensure that the system can be applied with low costs, few by-products and high efficiency on a daily basis, even at low pollutant concentrations.

Looking at the effect of the initial dye concentration, it is observed that a wide range of adsorbents can be used, with efficiencies of more than 90% even at high concentration values. In most cases, the increase of the dye concentration negatively influenced the removal efficiency. The investigated studies covered a concentration range from 3 to 1000 mg/L. In the studies, the removal time ranged from 5 min to 36 h. However, 100% efficiency was achieved in intervals of up to 5–60 min.

The removal of 16 anionic and cationic dyes was demonstrated. Among the anionic dyes, direct dyes are the most frequently tested, while Methylene Blue is the model dye for cationic dyes. Most of the studies have investigated the removal of dyes between pH 2 and 10. Having examined the chemistry of the solution, it can be concluded that anionic and cationic dyes behave differently in acidic and basic media. When designing the adsorption process, it is important to keep in mind the ionic nature of the dye, thus reducing the time required for the optimization study.

Through numerous examples of adsorbents, it has been observed that small amounts (as small as 0.05 g) have been found to remove dye with efficiencies greater than 85%. The conclusion of 14 scientific papers (shown in Table 3) is that as the amount of adsorbent increases, the removal efficiency of dyes increases and the maximum amount of bound substances decreases. Bearing in mind that the efficiency varied from 8 to up to 99% in the articles studied by reducing the particle size, it can be said that particle size is a highly influential factor. Therefore, in future research, if possible and feasible, it is important to increase surface area and porosity by reducing particle size. The effect of aqueous solution temperature (Table 5) was investigated between 288 and 348 K. Both endothermic and exothermic adsorption processes were observed. From a green chemistry point of view, the exothermic process is preferable, since no excess energy input is required by heating the system for optimal adsorption. It is observed that the dye does not affect the endothermic or exothermic nature of the process. Methylene Blue and Congo Red, with different adsorbents, showed both endothermic and exothermic characteristics. Temperature, in addition to adsorption efficiency, affects the nature and mechanism of adsorption.

Using the eluents shown in the last figure, it can be seen through examples that many adsorbents can be recycled over several cycles.

## Figures and Tables

**Table 3:** Results of various research regarding the effect of initial adsorbent dosage.

Adsorbent	Dyestuff	Adsorbent Dosage	Efficiency Range (%)	Quantity in Equilibrium Range (q_e_ mg/g)	Reference
walnut shell	Methylene Blue	0.5–2 g/L	-	178.93–47.51	[195]
magnetic alginate/rice husk bio-composite	Methylene Blue	0.1–1 g	15–89	338–145	[196]
Tunisian smectite clay	Cristal Violet	0.05–0.3 g/L	10–100	-	[197]
modified activated carbon (PABA@AC)	Malachite Green	10–50 mg	31.3–86.6	11.67–6.5	[198]
commercial natural activated plant-based carbon (CNAC)	Methylene Blue	0.5–1.5 g	46–78	-	[190]
commercial natural activated plant-based carbon (CNAC)	Eosin Yellow	0.5–1.5 g	51–70	-	[190]
commercial natural activated plant-based carbon (CNAC)	Rhodamine B	0.5–1.5 g	52–60	-	[190]
calcined eggshell	Remazol Brilliant Violet-5R	0.5–2 g	89.83–96.3	3.59–0.96	[134]
calcined eggshell	Remazol Red F3B	0.5–2 g	92–93.67	3.68–0.94	[135]
calcined eggshell	Remazol Blue RR	0.5–2 g	92–93.33	3.68–0.94	[135]
eggshell	Remazol Brilliant Violet-5R	0.5–2.5 g	74.67–93.85	2.96–0.75	[51]
activated carbon from lotus leaves	Methylene Blue	0.5–10 g/L	82.84–98.032	16.57–0.98	[192]
municipial solid waste compost ash	Reactive Red 198	0.5–2 g/L	79.25–92.92	-	[193]
natural clayey composite	Basic Navy Blue 2RN	0.2–1.2 g/50 mL	78–97	-	[199]
natural clayey composite	Drimaren Yellow CL-2R	0.2–1.2 g/50 mL	87–97	-	[199]
geopolymer	Methylene Blue (10^−5^ M)	0.05–0.1 g	79.8–85.6	-	[200]
mucilage of Salvia seeds	Cationic Blue 41	0.5–4 g/L	34.2–53.9	34.2–6.74	[201]
raw petroleum coke	Congo Red	4–24 g/L	~10–60	-	[202]
activated petroleum coke	Congo Red	4–24 g/L	~15–70	-	[202]

**Table 4:** Results of various research regarding the effect of adsorbent particle size.

Dyestuff	Adsorbent	Particle Size (μm)	Efficiency Range (%)	Quantity in Equilibrium Range (q_e_ mg/g)	Reference
Congo Red	cabbage waste powder	150–300 to 360–4750	75.95–8.03	-	[208]
Reactive Black 5	macadamia seed husks	150–300 to 2360–4750	98.9–33.2	-	[209]
Maxilon Blue GRL	coconut shell activated carbon	50, 75, and 106	-	~27.5–22.5–17.5	[205]
Direct Yellow DY 12	coconut shell activated carbon	50, 75, and 107	-	~5.5–4.5–3.5	[205]
Methylene Blue	*Cucumis sativus* peel waste	80–150, 150–200, and >200 BSS mesh	80.25–84.15–85.23	-	[210]
Crystal Violet	coffee husks	0.15–0.3 to 2.36–4.75 mm	96.082–89.854	-	[211]
Methylene Blue	clay3	177–250 to 400–840	99–86.4	-	[212]
